# Intramedullary Fibular Fixation in One-Stage Ankle Fracture Surgery With Soft Tissue Damage: A Study of 19 Cases

**DOI:** 10.7759/cureus.45599

**Published:** 2023-09-20

**Authors:** Mehmet Selçuk Saygılı

**Affiliations:** 1 Department of Orthopedics and Traumatology, University of Health Sciences, Prof. Dr. Cemil Taşcıoğlu City Hospital, Istanbul, TUR

**Keywords:** intramedullary nail, fibula, fibula nail, union, soft tissue injury, reduction quality, ulna nail, fibula fracture

## Abstract

This study investigates the efficacy of one-stage surgical intramedullary fibular fixation in managing ankle fractures with associated soft tissue damage. Ankle fractures, often encountered, can lead to complications when coupled with soft tissue injury. Traditional plate and screw fixation can exacerbate infection risks and reduce wound healing. To address this, a minimally invasive approach employing intramedullary fixation of the fibula has been proposed. This retrospective analysis, conducted between 2019 and 2021, explores cases of intramedullary fibular fixation for ankle fractures with stage 2-3 soft tissue injuries. A total of 19 patients were included in the study. The procedure involved either ulna intramedullary nails or locking screws. Results indicate that the approach led to successful union (100%), one superficial infection (5.26%), and no complication was observed. While limitations include the retrospective nature and small sample size, this study contributes valuable insights into the use of intramedullary fibular fixation in one-stage surgery for ankle fractures with concurrent soft tissue damage.

## Introduction

Ankle fractures are the most commonly encountered type of fractures [1]. When accompanied by soft tissue damage, these fractures exhibit increased complication rates [2]. In ankle fractures with associated soft tissue damage, the literature has reported that fixation of the fibula with plates and screws enhances the risk of infection and delays wound healing [3]. Therefore, in such fractures, some surgeons suggest a two-stage surgical approach [1,3,4]. Intramedullary fixation is a minimally invasive technique that reduces soft tissue trauma. Intramedullary fixation of the fibula is a well-known method for managing ankle fractures [5]. The advantages of this approach include reduced complication rates, accelerated healing, reduced infection risk, and blood loss [6-9]. This study aims to investigate cases in which intramedullary fibular fixation was preferred due to the minimally invasive nature of the procedure in the management of ankle fractures with associated soft tissue damage, treated through a one-stage surgical procedure, particularly in patients for whom the use of plate and screw fixation was not suitable because of soft tissue damage. Our hypothesis is that one-stage surgical intramedullary fibular fixation effectively reduces complications associated with ankle fractures with concurrent soft tissue damage.

## Materials and methods

**Figure 1:**
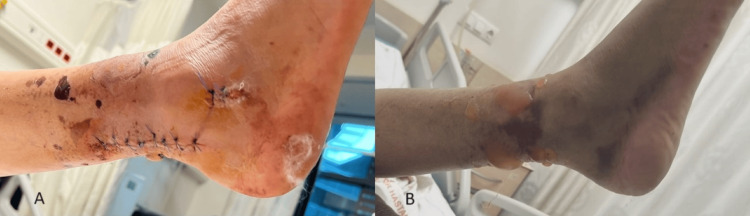
Ankle fracture with soft tissue injury (A) After one-stage surgery (posterolateral approach for posterior malleol fixation and lateral incision for intramedullary fibula fixation, (B) condition of the skin before the surgery

**Figure 2:**
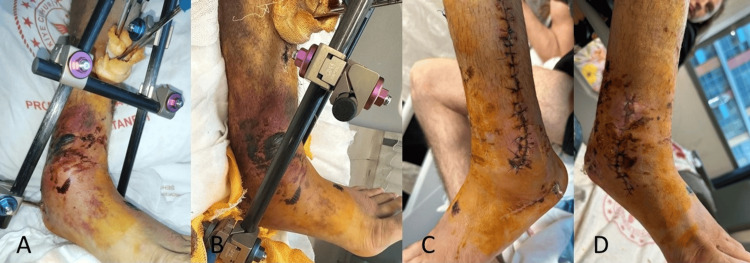
Two-stage surgery for ankle fractures (A, B) Anteromedial view of the injured ankle after first stage (application of external fixation), (C) lateral view of ankle after second stage (open reduction and fixation), (D) medial view of ankle after second stage (open reduction and fixation)

A retrospective analysis was conducted on patients treated for ankle fractures at the University of Health Sciences, Department of Orthopaedic and Traumatology, Prof. Dr. Cemil Taşcıoglu City Hospital between 2019 and 2021. Ethical approval was granted from the institutional review board (IRB) of Prof. Cemil Taşcıoğlu City Hospital - Istanbul (Approval no. 48670771-514, Date: 11/08/2022). Inclusion criteria were defined as follows: diagnosis of ankle fracture, intramedullary fibular fixation, age 18 and older, sufficient follow-up duration, the presence of stage 2-3 soft tissue injury according to the Oestern and Tscherne classification [10] of soft tissue injury in closed fractures (Figures 1A, 1B), this classification is based on energy transferred to the soft tissue. Grade 1: Superficial abrasion/contusion, Grade 2: Deep abrasions; impending compartment syndrome and Grade 3: Extensive skin contusion; myonecrosis; degloving; vascular injury; compartment syndrome. Exclusion criteria included pilon fractures, pediatric patients, patients undergoing a two-stage surgical approach (Figures 2A-2D) and patients with follow-up durations of less than six months. After screening, 33 patients who underwent intramedullary fibular fixation were identified. Nine pilon fractures, two pediatric patients, and three patients who underwent a two-stage surgical approach were excluded from the study. A total of 19 patients were included in the study.

**Figure 3:**
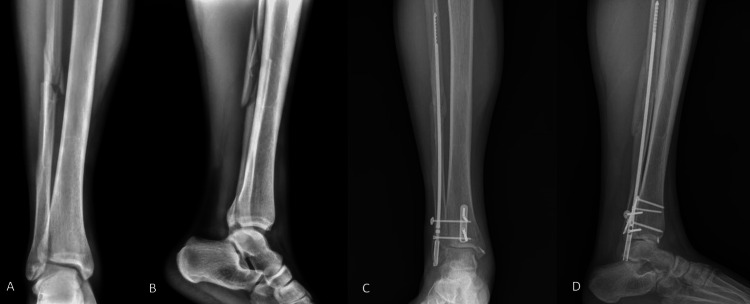
Nail fixation (A) Preoperative anteroposterior radiograph, (B) preoperative lateral radiograph, (C) postoperative (one year follow-up) anteroposterior radiograph, (D) postoperative (one year follow-up) lateral radiograph

**Figure 4:**
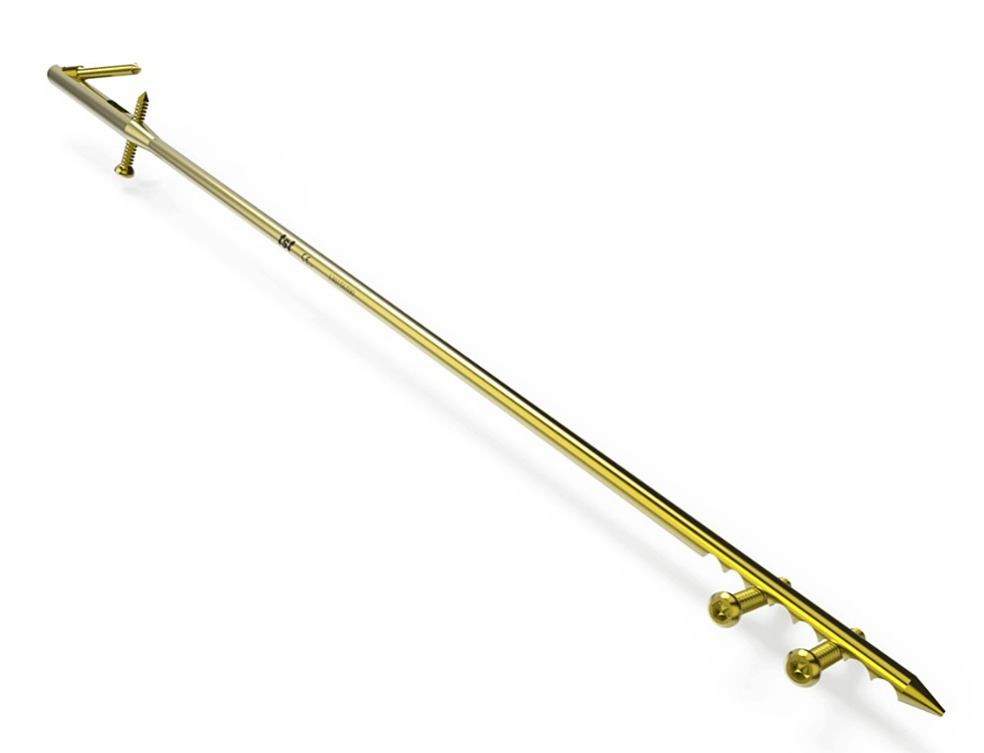
Ulna intramedullary nail

**Figure 5:**
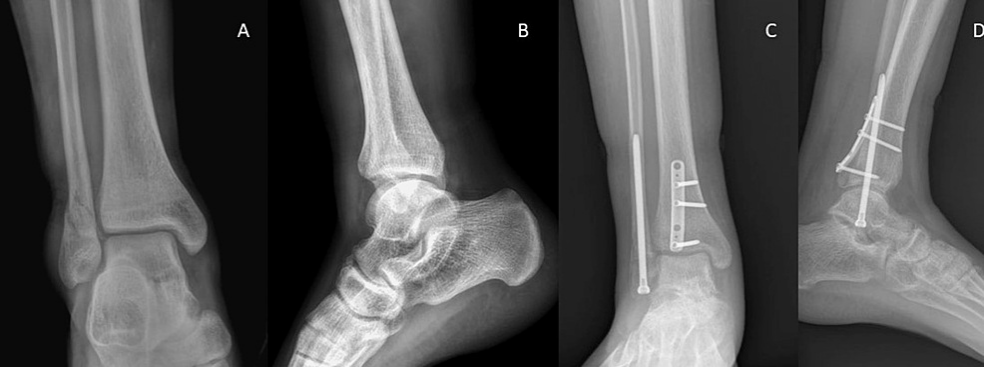
Screw fixation (A) Preoperative anteroposterior radiograph, (B) preoperative lateral radiograph, (C) postoperative (one year follow-up) anteroposterior radiograph, (D) postoperative (one year follow-up) lateral radiograph

**Figure 6:**
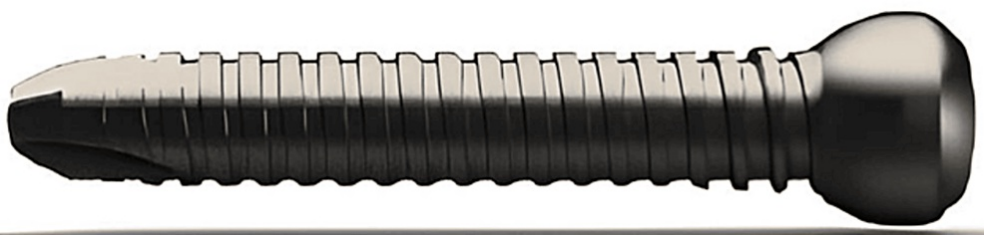
5.0mm full threaded nail locking screw

For fixation, ulna intramedullary nails (TST®, Turkey) were used in six patients (Figures 3A-3D, 4), while 5.0mm full threaded nail locking screws (Tasarımmed®, Turkey) were used in the remaining 13 patients (Figures 5A-5D, 6). The surgeries were performed by two different senior surgeons specialized in foot surgery. Cases with intramedullary screw fixation were operated on by one surgeon, while the other surgeon operated on cases with intramedullary nailing. Radiological images were evaluated by another senior surgeon.

All patients underwent surgery under spinal anesthesia. In all cases, 5 mm skin incision was made distally on the fibula. In cases with screws, one intramedullary screw was applied under fluoroscopy control after drilling at the tip of the fibula. In cases with nails, the fibula medulla was reamed by entering through the fibula tip, followed by the placement of an appropriate diameter and length intramedullary nail intramedullary. Then, two locking screws were applied distal to the fracture using an external guide. No locking screw was applied proximal to the fracture. Following the surgery, all patients were discharged with a short leg cast.

Patients were called in for follow-up on the 15th postoperative day. The short leg cast was removed, and ankle exercises were initiated. Patients were instructed to perform non-weight bearing walking. At week 4, patients were allowed to bear weight as tolerated. After the sixth week, full weight-bearing was allowed upon clinical and radiological evidence of union.

Patient-related data including age, gender, fracture side, fracture type according to Lauge-Hansen [11], fracture type according to Weber [12], complications, time to union, and postoperative reduction quality were recorded using the evaluation system described by McLennan and Ungersma [13] and were statistically analyzed.

Patient data were analyzed using IBM Statistical Package for the Social Sciences (SPSS) for Windows 25.0 software (IBM Corp., Armonk, NY). Categorical data were presented as frequencies and percentages, while continuous data were presented as means and standard deviations.

## Results

The mean age of the patients included in the study was 40.21 ± 20.01 years. All patients underwent surgery within an average of three days (2-5 days) after trauma. The gender distribution was 11 females (57.9%) and eight males (42.1%). Regarding the distribution by fracture side, 52.6% of patients had fractures on the right side, and 47.4% had fractures on the left side. In terms of treatment methods, ulna nails were used in six patients (31.6%), while intramedullary screws were preferred in 13 patients (68.4%) (Table 1).

**Table 1:** Descriptive data

	Implant type	Age (years)	Gender	UnIon tIme (days)	ReductIon qualIty	EtIology	Lauge-Hansen ClassIfIcatIon	DenIs Weber ClassIfIcatIon
Case #1	Nail	18	Female	122	Fair	Fall from height	PER	C
Case #2	Nail	61	Male	62	Good	Car accident	PAB	B
Case #3	Nail	73	Male	66	Poor	Car accident	PER	C
Case #4	Nail	26	Male	87	Good	Simple fall	PER	C
Case #5	Nail	29	Male	57	Good	Fall from height	PER	C
Case #6	Nail	53	Male	73	Good	Car accident	PER	C
Case #7	Screw	51	Female	56	Good	burkulma	SAD	A
Case #8	Screw	19	Male	56	Good	Gunshot	SAD	A
Case #9	Screw	21	Male	63	Good	Car accident	SER	B
Case #10	Screw	54	Female	94	Good	Simple fall	SER	B
Case #11	Screw	22	Female	42	Good	Simple fall	SER	B
Case #12	Screw	37	Male	83	Good	Car accident	SAD	B
Case # 13	Screw	52	Female	62	Fair	Simple fall	SAD	B
Case #14	Screw	28	Male	54	Fair	Car accident	SAD	B
Case #15	Screw	75	Female	61	Good	Simple fall	SAD	A
Case #16	Screw	75	Female	64	Good	Simple fall	SAD	A
Case #17	Screw	31	Male	64	Good	Car accident	SER	A
Case #18	Screw	20	Male	76	Good	Car accident	SER	B
Case #19	Screw	19	Female	55	Good	Fall from height	PER	C

Reduction was determined based on the method described by McLennan and Ungersma. The good reduction was defined as the fibula being out to a length; <2 mm posterior displacement; and <1 mm increase in medial clear space. A fair reduction was described as fibular shortening of <2 mm; 2 mm-4 mm posterior displacement; 2 mm lateral displacement; and 1 mm-3 mm increase in medial clear space. A poor reduction was defined as fibular shortening >2 mm; >4 mm posterior displacement; >2 mm lateral displacement; and >3 mm increase in medial clear space. Only one superficial infection was observed in one patient Tscherne's grade 3 injury and none in the grade 2 injury. When assessing the reduction quality after intramedullary fibular fixation, good results were achieved in 78.9% of patients, moderate in 15.8%, and poor in 5.3% of patients.

In terms of fracture types, according to the Lauge-Hansen classification, 26.3% of patients had supination-external rotation (SER) type fractures, 31.6% had pronation-external rotation (PER) type fractures, 36.8% had supination-adduction (SAD) type fractures, and 5.3% had pronation-abduction (PAB) type fractures. According to the Weber classification, 26.3% of patients had Weber A fractures, 42.1% had Weber B fractures, and 31.6% had Weber C fractures.

In 15.8% of patients, a syndesmosis screw was also used in addition to intramedullary nailing. The average time to union was 64.78 ± 23.3 days. Union was observed in all patients. Superficial infection was observed in a single patient. No intervention or hospitalization was needed for infection. Just oral anti-biotherapy was given. No additional complications were detected.

## Discussion

Our study revealed that intramedullary fixation for fibular fractures in one-stage surgery effectively promotes union and achieves low rates of infection and complications in cases of soft tissue injury associated with ankle fractures. Additionally, to the best of our knowledge, only one study evaluates fibular intramedullary nailing in ankle fractures with soft tissue damage [14]. However, this study included diabetic foot patients, individuals with chronic skin issues, and open fractures [14]. To the best of our knowledge, our study is the first investigation in the literature focusing solely on closed fractures with soft tissue damage sustained after trauma.

Open reduction and internal fixation are acknowledged treatment option in cases of ankle fracture with associated soft tissue damage and without major soft tissue complications [15]. Alternatively, two-stage surgery or external fixation can be considered based on the condition of the soft tissues [1,3,4]. Literature on surgical timing generally suggests that these fractures should be operated on within the first 8 hours or after five to six days. However, Saithna et al. [16] stated that delaying surgery is unnecessary and increases soft tissue complications. In Saithna et al.'s study [16], all infected patients had swollen soft tissues upon hospital admission. Tanoğlu et al. [1] compared two-stage and one-stage ankle fracture surgeries and found that all patients with Tscherne grade 2-3 soft tissue damage were in the two-stage surgery group. However, intramedullary nailing of the fibula in ankle fractures with soft tissue damage could have the advantage of being performed in a one-stage procedure [2]. According to the results of this study, we believe that one-stage treatment with intramedullary fibula fixation can be applied in ankle fractures with accompanying soft tissue damage.

Publications report that intramedullary fixation of the fibula reduces the risk of infection compared to plate and screw fixation [13]. Lee et al. [6], in their study evaluating the five-year outcomes of open AO type B2 fractures, did not encounter any infections in the group treated with intramedullary fixation, while four (18.2%) patients in the other group developed postoperative infections. White et al. [9], in their study comparing intramedullary nail and plate and screw fixation in elderly patients, found a lower infection rate in the nail group. Morelli et al. [17] also mentioned that fibular fixation with a rush pin reduces the risk of infection compared to plate and screw fixation. Current publications even report infection rates as low as 0% [6,18]. Only one patient (5.3%) developed a postoperative infection in our study. Intramedullary fibular nailing may minimize surgical soft tissue damage, possibly explaining the lack of an increase in infection rates in ankle fractures with soft tissue damage.

The quality of reduction in ankle fractures is important for preventing the development of osteoarthritis [19]. In studies evaluating radiological reduction quality, Bugler et al. [20] reported excellent results in 77 (96%) patients and fair results in three (4%) patients. Similarly, Al-Obaidi et al. [21] reported excellent results in 33 (87%) patients, fair results in three (8%), and poor results in two (5%). The reduction quality ratios in our study are consistent with the literature; however, the lower percentage of excellent results in our study could be attributed to the use of multiple implant types and not employing a specific fibular nail. Additionally, the small sample size might have contributed to this outcome. On the other hand, the reduction rates were within an acceptable range of 95%.

In the literature, complications related to fibular nails include nail breakage, guidewire breakage, and misplacement of locking screws [22]. Goss et al. [23] mentioned in cadaver studies that peroneal tendons and the superficial peroneal nerve are at risk in nail fixation of distal fibula fractures. Removal of the locking screw may also be required in the fibular intramedüller fixation [7]. However, these complications were not observed in our study. It is important to remember that these complications are associated with fibular nailing and can potentially occur.

Several limitations exist in our study. These include its retrospective nature, small sample size, lack of control group and single-center design. The use of different fixation materials in patients is one limitation. Another limitation is the lack of evaluation of postoperative functional outcomes. Heterogeneity in injury mechanisms and fracture types could be considered as additional limitations. Multi-center, prospective randomized controlled trials with larger sample sizes could provide more comprehensive information. Despite these limitations, our study, being the first to evaluate fibular intramedullary nailing in the surgery of ankle fractures with soft tissue damage, will contribute to the literature.

## Conclusions

In conclusion, intramedullary fixation for fibular fractures with soft tissue damage in one-stage surgery effectively provides union and achieves low rates of infection. Also, one-stage surgery with intramedullary fibular fixation effectively reduces complications and promotes healing in ankle fractures with soft tissue damage. Multi-center, prospective randomized controlled trials with larger sample sizes could provide more comprehensive information.
